# Human complement receptor type 1 (CR1) protein levels and genetic variants in chronic Chagas Disease

**DOI:** 10.1038/s41598-017-18937-z

**Published:** 2018-01-11

**Authors:** Thaisa Lucas Sandri, Kárita Cláudia Freitas Lidani, Fabiana Antunes Andrade, Christian G. Meyer, Peter G. Kremsner, Iara J. de Messias-Reason, Thirumalaisamy P. Velavan

**Affiliations:** 1 Laboratory of Molecular Immunopathology, Federal University of Paraná, Curitiba, Brazil; 2 Institute of Tropical Medicine, University of Tübingen, Tübingen, Germany; 3 Faculty of Medicine, Duy Tan University, Da Nang, Vietnam; 4 Vietnamese - German Center for Medical Research, Hanoi, Vietnam

## Abstract

Complement is an essential element in both innate and acquired immunity contributing to the immunopathogenesis of many disorders, including Chagas Disease (CD). Human complement receptor 1 (CR1) plays a role in the clearance of complement opsonized molecules and may facilitate the entry of pathogens into host cells. Distinct *CR1* exon 29 variants have been found associated with CR1 expression levels, increased susceptibility and pathophysiology of several diseases. In this study, CR1 plasma levels were assessed by ELISA and *CR1* variants in exon 29 by sequencing in a Brazilian cohort of 232 chronic CD patients and 104 healthy controls. CR1 levels were significantly decreased in CD patients compared to controls (p < 0.0001). The *CR1* rs1704660*G*, rs17047661*G* and rs6691117*G* variants were significantly associated with CD and in high linkage disequilibrium. The CR1*AGAGTG haplotype was associated with *T. cruzi* infection (p = 0.035, OR 3.99, CI 1.1-14.15) whereas CR1*AGGGTG was related to the risk of chagasic cardiomyopathy (p = 0.028, OR 12.15, CI 1.13-113). This is the first study that provides insights on the role of CR1 in development and clinical presentation of chronic CD.

## Introduction

Chagas Disease (CD) is a neglected infectious disease caused by the intracellular protozoan parasite *Trypanosoma cruzi*. CD affects more than five million people in Latin America and another 20 million are at risk of acquiring the infection^1^. Approximately 300,000 new cases are reported to occur each year, and approximately 21,000 patients die annually^2^. Human migration has contributed substantially to the current global scenario of CD increasing the number of cases in non-endemic countries with epidemiological, economic and social implications, which challenge control of the spread of *T. cruzi*^3^. The annual cost due to CD globally is US$ 7.19 billion and has a lifetime cost of US$ 27,684 per infected individual^4^.

Although most individuals infected with *T. cruzi* remain asymptomatic all lifelong, approximately 2–5% of infected individuals progress each year to a symptomatic form of the disease, developing either chronic chagasic cardiomyopathy (CCC) or digestive megasyndromes, or both^5^. About 10% of patients develop lethal cardiomyopathy, with heart transplantation remaining the ultimate treatment available^6^. CCC is an inflammatory condition characterized by intense Th1-type immune response^7^. After initial infection, Th1 proinflammatory cytokines are produced and this production continues along chronic phase, likely due to parasite persistence among others^6^. Persistent Th1-type response can lead to cardiac commitment, starting with myocarditis and then progressing to CCC^8^.

After transmission of the pathogen by the triatomine insect vector (subfamily Triatominae), *T. cruzi* uses several mechanisms to escape host immune responses, among which is the evasion from complement attack^9–11^. The infective form of *T. cruzi*, the metacyclic trypomastigotes, can invade almost all nucleated cells^12^ by involving a wide diversity of receptors such as kinins, receptor tyrosine kinases, transforming and epidermal growth factor receptors, the lectin receptor Gallectin-3, fibronectin, and Toll-like receptors^13,14^. In addition to these receptors, *T. cruzi* utilizes the complement molecules such as C1q to promote C1-dependent phagocytosis and the lectin proteins mannose-binding lectin (MBL) and ficolin-2 to evade host immune attack and promote infection^10,15^. Evans-Osses and collaborators (2014) suggested that the deposition of MBL on *T. cruzi* parasite surface plays a role in the infection process, while the parasite deactivates the lectin complement pathway^16^, which ultimately could favor *T. cruzi* cell internalization mediated by receptors for both molecules, including CR1.

The complement system is essential in both innate and acquired immunity^17^, contributing to the immunopathogenesis of a variety of diseases, including CD^10,11,18,19^. CR1, or CD35, is a multi-functional polymorphic glycoprotein, which occurs as a soluble or transmembrane protein expressed on peripheral blood cells including monocytes and erythrocytes, natural killer cells as well as on B and T cells^17,20^. CR1 is known to enhance phagocytosis of particles opsonized with C3b, C4b, C1q, MBL, and ficolin-2 as well as to facilitate the clearance of immune complexes by binding to CR1 on erythrocytes and macrophages for further disposal^21,22^. The *CR1* gene is located on chromosome 1q32.2 (OMIM 120620) and belongs to the Regulator of Complement Activation family, which is characterized by small consensus repeats, also known as complement control protein repeats^17,22^. Genetic variability may influence CR1 expression including its molecular weight and the density of CR1 molecules on cell surfaces^22,23^.

It has been demonstrated that CR1 is involved in the pathogenesis of several of infectious diseases either by facilitating pathogens entry into host cells in some cases or by down-modulating complement activation in others^24,25^. CR1 was shown to mediate immune opsonization of *Leishmania* amastigotes and promastigotes^26,27^, *Plasmodium falciparum*^28^, *Mycobacterium tuberculosis*^29^, *M. leprae*^30^, HIV^31,32^, SARS-CoV^33^, adenovirus^34^ hepatitis C virus^31^ and West Nile Virus^35^.

Besides the role of CR1 in facilitating the entry of intracellular pathogens into host cells, CR1 protein levels were shown to be associated with the pathogenesis of different diseases including malaria^28^, tuberculosis^36^, lepromatous leprosy^37^, severe acute respiratory syndrome^33^, chronic liver diseases^38^, HIV infection among others^39^. The *CR1* genetic variants in exon 29 evaluated in this study (rs17259045, rs41274768, rs17047660, rs17047661, rs4844609 and rs6691117) are of particular interest since all are non-synonymous variants (https://www.ensembl.org) that are situated at the binding site for C1q, ficolins and MBL having thereby potential to influence the complement induced phagocytosis^21,22^. The present study aimed to assess if the genetic variants in exon 29 and CR1 levels are associated with development and clinical presentation of chronic CD.

## Results

### CR1 plasma levels

**Figure 1:**
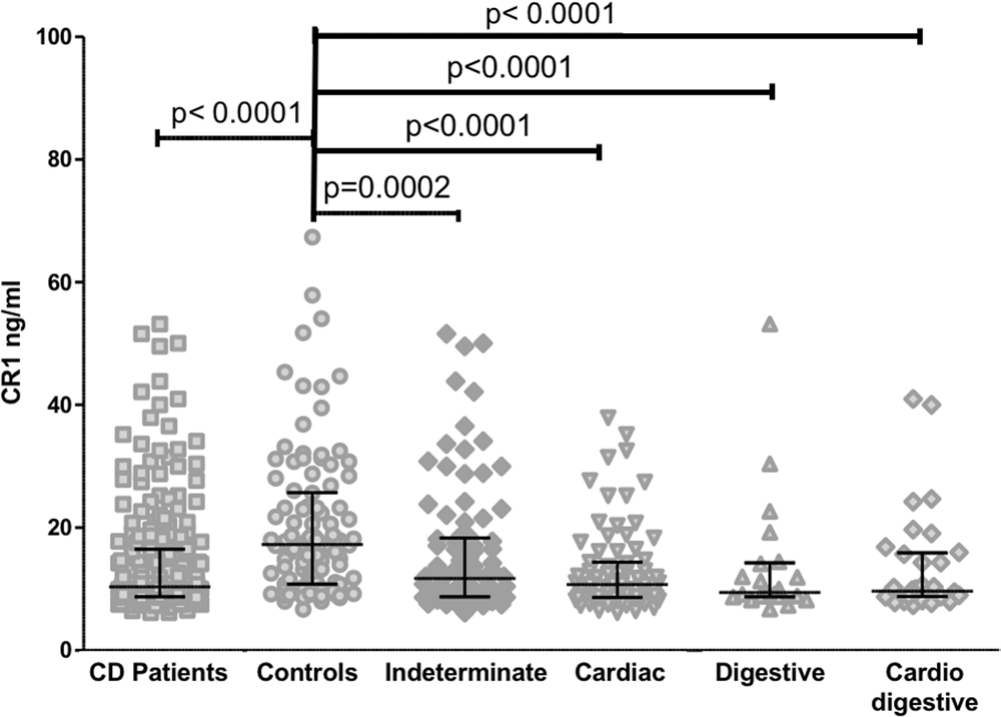
CR1 plasma levels in patients with CD and controls.

CR1 plasma levels were significantly lower in CD patients compared to controls (p < 0.0001), (Fig. 1). When comparing controls to each clinical form separately, statistical differences were also observed for CR1 levels between controls and the indeterminate form (p = 0.0002), cardiac form (p < 0.0001), digestive form (p < 0.0001), and cardiodigestive form (p < 0.0001) (Fig. 1). Comparison of CR1 levels between asymptomatic (indeterminate form) and symptomatic patients showed no statistical difference.

### Association of CR1 variants with Chagas disease

The distribution of *CR1* genotypes in controls was in Hardy-Weinberg equilibrium (p > 0.05), in patients with chronic CD three SNPs (rs17047660, rs17047661, rs4844609) were not in HW equilibrium, which may be due to disease association. The frequencies of *CR1* variants rs17047660*G* (p = 0.02, OR 5.06, 95%CI 1.17-21.81), rs17047661*G* (p = 0.0042, OR 3.03, 95%CI 1.34-9.9) and rs6691117*G* (p = 0.015, OR 1.6, 95%CI 1.09-2.35) were significantly higher in CD patients compared to controls (Table 1). Also, the frequencies of the *CR1* genotypes rs17047661*AG* and rs17047661*GG* (p = 0.015, OR 3.0, 95%CI 1.25-7.49) and rs6691117*AG* and rs6691117*GG* (p = 0.004, OR 2.2, 95%CI 1.26-3.53) were significantly higher in chronic CD patients than in controls (Table 1).

**Table 1:** *CR1* genotypes and allele frequencies in patients with chronic CD and healthy controls.

*CR1* genetic variants		Control n = 102 (%)	CD Patients n = 220 (%)	Indeter minate n = 87 (%)	Cardiac n = 77 (%)	Digestive n = 19 (%)	Cardio digestive n = 31 (%)	CD Patients vs. Controls p value; OR [95% CI]	Indeterminate vs. Controls p value; OR [95% CI]	Cardiac vs. Controls p value; OR [95% CI]	Digestive vs. Controls p value; OR [95% CI]
rs17259045A/G	*AA*	82 (80)	190 (87)	75 (86)	69 (90)	16 (84)	25 (81)				
	*AG*	20 (20)	27 (12)	11 (13)	7 (9)	3 (16)	5 (16)	NS	NS	NS	NS
	*GG*	0	3 (1)	1 (1)	1 (1)	0	1 (3)				
	A*	184 (90)	407 (92)	161 (92)	145 (94)	35 (92)	55 (89)	NS	NS	NS	NS
	*G*	20 (10)	33 (8)	13 (8)	9 (6)	3 (8)	7 (11)				
rs41274768G/A	*GG*	98 (96)	198 (90)	80 (92)	70 (91)	16 (84)	27 (87)				
	*GA*	4 (4)	22 (10)	7 (8)	7 (9)	3 (16)	4 (13)	NS	NS	NS	NS
	*AA*	0	0	0	0	0	0				
	G*	200 (98)	418 (95)	167 (96)	147 (95)	35 (92)	58 (93)	NS	NS	NS	NS
	*A*	4 (2)	22 (5)	7 (4)	7 (5)	3 (8)	4 (7)				
rs17047660A/G	*AA*	100 (98)	202 (92)	81 (93)	70 (91)	19 (100)	28 (90)				
	*AG*	2 (2)	15 (7)	5 (6)	6 (8)	0	2 (6)	NS	NS	NS	NA
	*GG*	0	3 (1)	1 (1)	1 (1)	0	1 (3)				
	A*	202 (99)	419 (95)	167 (96)	146 (95)	38 (100)	58 (94)	p = 0.02; 5.06 [1.17–21.81]	NS	NS	NA
	*G*	2 (1)	21 (5)	7 (4)	8 (5)	0	4 (6)				
rs17047661A/G	*AA*	95 (93)	183 (83)	76 (87)	61 (79)	16 (84)	27 (87)				
	*AG*	7 (7)	31 (14)	9 (10)	13 (17)	3 (16)	3 (10)	p = 0.015; 3.05 [1.25–7.49]^1^	NS	p = 0.023; 3.74 [1.19–11.72]^1^	NS
	*GG*	0	6 (3)	2 (2)	3 (4)	0	1 (3)				
	A*	197 (97)	397 (90)	161 (93)	135 (88)	35 (92)	57 (92)	p = 0.0042; 3.03 [1.34–9.9]	NS	p = 0.0017; 3.96 [1.62–9.68]	NS
	*G*	7 (2)	43 (10)	13 (7)	19 (12)	3 (8)	5 (8)				
rs4844609T/A	*TT*	100 (98)	213 (97)	84 (97)	75 (97)	18 (95)	30 (97)				
	*TA*	2 (2)	4 (2)	3 (3)	1 (1.5)	0	0	NS	NS	NS	NS
	*AA*	0	3 (1)	0	1 (1.5)	1 (5)	1 (3)				
	T*	202 (99)	430 (98)	171 (98)	151 (98)	36 (95)	60 (97)	NS	NS	NS	NS
	*A*	2 (1)	10 (2)	3 (2)	3 (2)	2 (5)	2 (3)				
rs6691117A/G	*AA*	62 (61)	99 (45)	36 (41)	39 (51)	7 (37)	16 (52)				
	*AG*	33 (32)	99 (45)	43 (49)	31 (40)	8 (42)	12 (39)	p = 0.004; 2.22 [1.26–3.53]^1^	p = 0.006; 2.34 [1.28–4.27]^1^	NS	NS
	*GG*	7 (7)	22 (10)	8 (9)	7 (9)	4 (21)	3 (10)				
	A*	157 (77)	297 (68)	115 (66)	109 (71)	22 (58)	44 (71)	p = 0.015;1.60 [1.09–2.35]	p = 0.02; 1.71 [1.09–2.69]	NS	p = 0.025; 2.42 [1.18–5.0]
	*G*	47 (23)	143 (32)	59 (34)	45 (29)	16 (42)	18 (29)				

NA: Not applicable, NS: Not significant, *Major allele in the investigated population.

When analyzing CD patients according to their clinical presentation in relation to controls, the rs6691117*G* allele occurred more frequently among asymptomatic indeterminate form of CD (p = 0.02, OR 1.7, 95%CI 1.09-2.69) and in patients presenting with the digestive form of CD (p = 0.025, OR 2.4, 95%CI 1.18-5.0) (Table 1). In addition, carriers of the *G* allele (rs6691117*AG* and rs6691117*GG*) were rather present among asymptomatic patients than in controls (p = 0.006, OR 2.3, 95%CI 1.28-4.27) (Table 1). A significant association with the cardiac form was found also for the minor *G* allele of rs17047661 (p = 0.017, OR 3.9, 95%CI 1.62-9.68) and for *G* carriers (*AG* and *GG*) (p = 0.023, OR 3.7, 95%CI 1.19-11.72) (Table 1).

Patients with cardiomyopathy, graded according to the classification of cardiomyopathy as outlined in the Methods section, were compared to asymptomatic patients. The rs17047661*G* allele occurred more frequently in patients with cardiomyopathy without ECHO alteration (p = 0.028, OR 2.8, 95%CI 1.14-7.16) and in patients with cardiomyopathy without heart failure (p = 0.0065, OR 2.8, 95%CI 1.33-6.02) than in asymptomatic patients. Both rs17047661*AG* and rs17047661*GG* genotypes were observed more frequently among patients with cardiomyopathy without ECHO alteration (p = 0.031, OR 3.3, 95%CI 1.11-9.75) and in patients with cardiomyopathy without heart failure (p = 0.02, OR 1.7, 95%CI 1.08-2.79) than in patients without overt symptoms (Table 2).

**Table 2:** *CR1* genotypes and allele frequencies in patients with chronic CD based on cardiac impairment.

*CR1* genetic variants		Control n = 102 (%)	Indeter minate n = 87 (%)	Without ECHO alteration n = 24 (%)	With ECHO alteration n = 74 (%)	Without Heart Failure n = 51 (%)	Heart Failure n = 47 (%)	Without ECHO alteration vs. Indeterminate p value OR [95%CI]	Without Heart Failure vs. Indeterminate p value OR [95%CI]	Heart Failure vs. Without Heart Failure p value OR [95%CI]
rs17259045*A/G*	*AA*	82 (80)	75 (86)	21 (88)	63 (85)	45 (88)	39 (83)			
	*AG*	20 (20)	11 (13)	1 (4)	11 (15)	4 (8)	8 (17)	NS	NS	NS
	*GG*	0	1 (1)	2 (8)	0	2 (4)	0			
	A*	184 (90)	161 (92)	43 (90)	137 (93)	94 (92)	86 (91)	NS	NS	NS
	*G*	20 (10)	13 (8)	5 (10)	11 (7)	8 (8)	8 (9)			
rs41274768*G/A*	*GG*	98 (96)	80 (92)	23 (96)	66 (89)	45 (88)	44 (94)			
	*GA*	4 (4)	7 (8)	1 (4)	8 (11)	6 (12)	3 (6)	NS	NS	NS
	*AA*	0	0	0	0	0	0			
	G*	200 (98)	167 (96)	47 (98)	140 (95)	96 (94)	91 (97)	NS	NS	NS
	*A*	4 (2)	7 (4)	1 (2)	8 (5)	6 (6)	3 (3)			
rs17047660*A/G*	*AA*	100 (98)	81 (93)	21 (88)	67 (91)	44 (86)	44 (94)			
	*AG*	2 (2)	5 (6)	3 (13)	5 (7)	7 (14)	1 (2)	NS	NS	NS
	*GG*	0	1 (1)	0	2 (3)	0	2 (4)			
	A*	202 (99)	167 (96)	45 (94)	139 (94)	95 (93)	89 (95)	NS	NS	NS
	*G*	2 (1)	7 (4)	3 (6)	9 (6)	7 (7)	5 (5)			
rs17047661*A/G*	*AA*	95 (93)	76 (87)	16 (69)	63 (85)	35 (69)	44 (93)			
	*AG*	7 (7)	9 (10)	7 (25)	8 (11)	13 (25)	2 (4)	p = 0.031; 3.30 [1.11–9.75]^1^	p = 0.02; 1.74 [1.08–2.79]^1^	p = 0.007; 0.15 [0.03–0.60]^1^
	*GG*	0	2 (2)	1 (6)	3 (4)	3 (6)	1 (2)			
	A*	197 (97)	161 (93)	39 (81)	134 (91)	83 (81)	90 (94)	p = 0.028; 2.85 [1.14–7.16]	p = 0.0065; 2.83 [1.33–6.02]	p = 0.0017; 0.19 [0.06–0.59]
	*G*	7 (2)	13 (7)	9 (19)	14 (9)	19 (19)	4 (4)			
rs4844609*T/A*	*TT*	100 (98)	84 (97)	24 (100)	71 (96)	51 (100)	44 (94)			
	*TA*	2 (2)	3 (3)	0	1 (1)	0	1 (2)	NA	NA	NA
	*AA*	0	0	0	2 (3)	0	2 (4)			
	T*	202 (99)	171 (98)	48 (100)	143 (97)	102 (100)	89 (95)	NA	NA	NA
	*A*	2 (1)	3 (2)	0	5 (3)	0	5 (5)			
rs6691117*A/G*	*AA*	62 (61)	36 (41)	12 (50)	40 (54)	24 (47)	28 (60)			
	*AG*	33 (32)	43 (49)	9 (37)	28 (38)	20 (39)	17 (36)	NS	NS	NS
	*GG*	7 (7)	8 (9)	3 (13)	6 (8)	7 (14)	2 (4)			
	A*	157 (77)	115 (66)	33 (69)	108 (73)	68 (67)	73 (78)	NS	NS	NS
	*G*	47 (23)	59 (34)	15 (31)	40 (27)	34 (33)	21 (22)			

NA: Not applicable, NS: Not significant, ^*^Major allele in the investigated population.

Comparing patients with cardiomyopathy and considering the different stages of cardiac pathology, the rs17047661*G* alleles (p = 0.0017, OR 0.19, 95%CI 0.06-0.59) and rs17047661*AG* and rs17047661*GG* genotypes (p = 0.007, OR 0.15, 95%CI 0.03-0.60) were more frequent in patients without heart failure than in those with heart failure (Table 2).

### Association of CR1 haplotypes with Chagas disease

**Figure 2:**
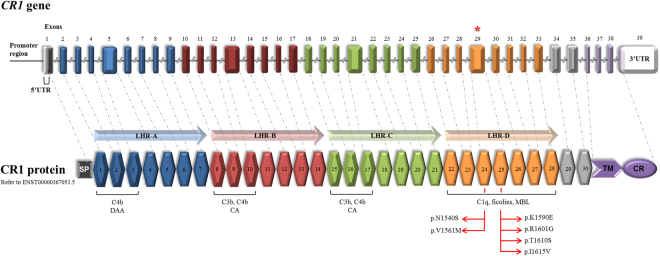
Diagrammatic representation of the *CR1* locus. The *CR1* locus based on CR1-205 transcript (ENST00000367053.5). Colored boxes represent exons, which encode a specific protein domain. The CR1 protein is composed of 30 short consensus repeats (SCR). Among them, 28 repeats are arranged in four long homologous repeats (LHR-A: 1-7, LHR-B: 8-14, LHR-C: 15-21 and LHR-D: 22-28). The first three SCRs of LHR A, B and C are required for complement binding (C3b and C4b) and also for decayed accelerating activity (DAA) or cofactor activity (CA), while LHR-D binds C1q, ficolins and mannose binding lectin (MBL). The connecting lines indicate representative exons coding specific SCRs sequences, signal peptide (SP), transmembrane domain (TM) and cytoplasmic region (CR). Six CR1 genetic variants analyzed are located in exon 29* and positioned in SCRs 24 and 25. The amino acid substitutions are indicated by red arrows. Exons are drawn to scale and introns are truncated.

**Figure 3:**
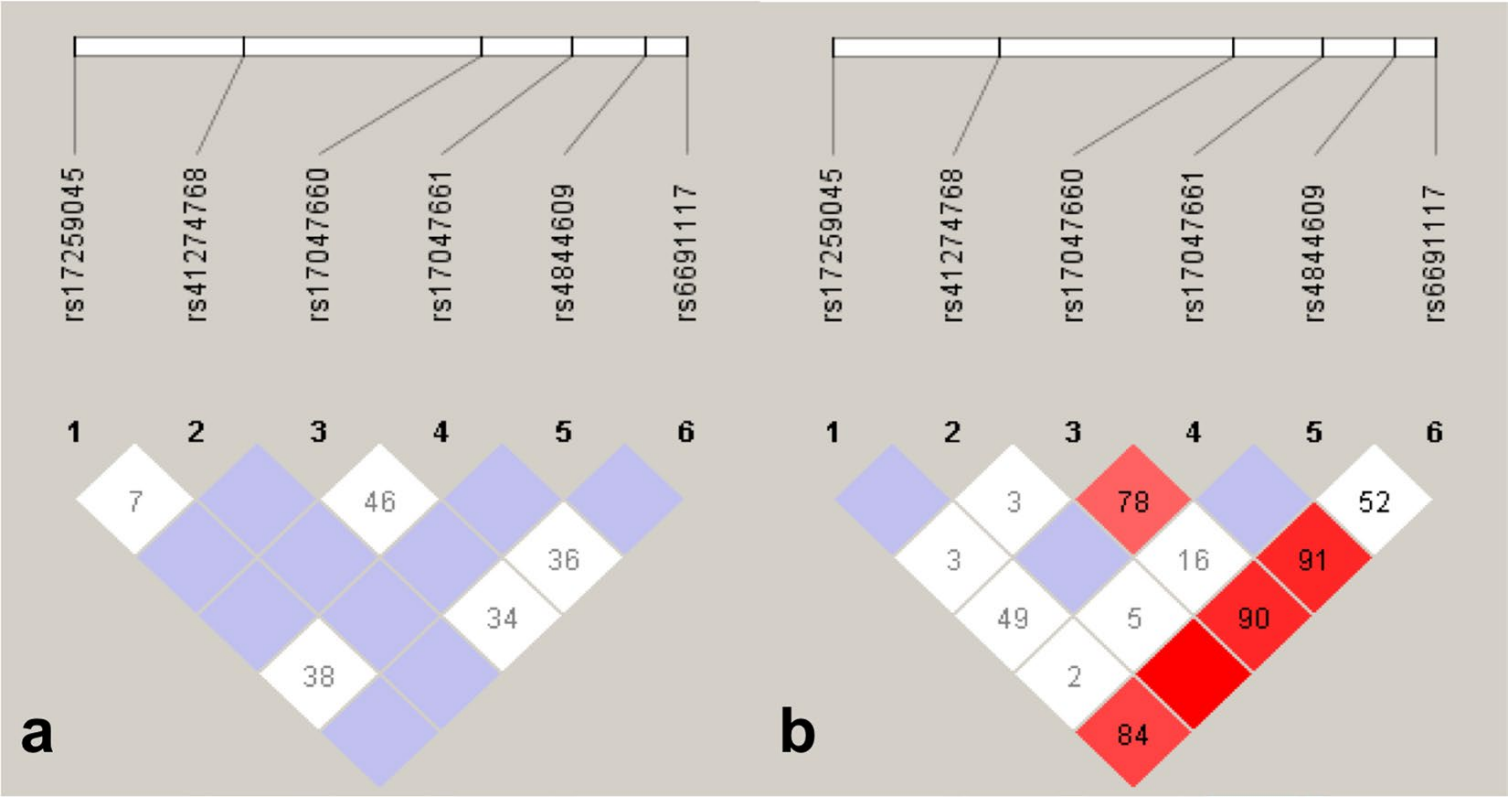
Linkage disequilibrium (LD) of the investigated exon 29 *CR1* variants. LD was calculated based on the data for controls (**a**) and patients with chronic CD (**b**), being the pairwise correlation coefficient values (D’) between tag SNPs referred by numbers inside the squares that show the amount of LD between two SNPs. Red, purple, and white squares represent high, medium and low levels of LD, respectively. Relative position of SNPs on *CR1* gene are indicated on the abscissas.

A total of 15 *CR1* haplotypes were observed, they were reconstructed from the six *CR1* variants (rs17259045, rs41274768, rs17047660, rs17047661, rs4844609, rs6691117) investigated in the study (Fig. 2). The frequency of CR1*AGAGTG haplotype was significantly increased among CD patients (p = 0.035, OR 3.9, 95%CI 1.10-14.15), in patients with cardiomyopathy without ECHO alteration (p = 0.03, OR 5.5, 95%CI 1.17-25.8), and in cardiomyopathy patients without heart failure (p = 0.005, OR 7.7, 95%CI 1.84-32.7) than among controls. In addition, CR1*AGGGTG was significantly associated with cardiomyopathy (p = 0.028, OR 12.1, 95%CI 1.3-113) and with the absence of heart failure (p = 0.037, OR 11.1, 95%CI 1.15-107) in comparison to controls (Table 3). Linkage disequilibrium (LD) patterns of the *CR1* variants are given in Fig. 3. Strong LD was observed only in chronic CD patients. The LD plot indicates that rs17259045, rs41274768, rs17047660, and rs17047661 were in strong LD with rs6691117; therefore, rs17047660 was also in strong LD with rs17047661.

**Table 3:** Reconstructed CR1 haplotypes among CD patients and controls.

CR1 haplotypes (+4659/+4721/+4808/+4841/+4868/+4883)	Controlx n = 204 (%)	CD Patient n = 440 (%)	Indetermi nate n = 174 (%)	Cardiac n = 154 (%)	Digestive n = 38 (%)	Cardio digestive n = 62 (%)	Without ECHO alteration n = 48 (%)	With ECHO alteration n = 148 (%)	Without Heart Failure n = 102 (%)	Heart Failure n = 94 (%)	Patient vs. Control p value;OR [95% CI]	Cardiac vs. Control p value; OR [95% CI]	Without ECHO Alteration vs. Control p value; OR [95% CI]	Without Heart Failure vs. Control p value; OR [95% CI]
CR1*AGAATA	131 (64.2)	253 (57.5)	99 (56.9)	94 (61)	17 (44.7)	37 (59.7)	28 (58)	92 (62.1)	59 (57.8)	61 (64.9)	NS	NS	NS	NS
CR1*AGAATG	39 (19)	81 (18.5)	39 (22.4)	22 (14.3)	10 (26.3)	9 (14.5)	5 (10.4)	20 (13.5)	10 (9.8)	15 (16)	NS	NS	NS	NS
CR1*GGAATA	20 (9.8)	31 (7)	12 (6.8)	9 (5.8)	3 (7.9)	6 (9.7)	5 (10.4)	10 (6.8)	8 (7.8)	7 (7.4)	NS	NS	NS	NS
CR1*AAAATG	4 (2)	18 (4.1)	7 (4)	5 (3.2)	3 (7.9)	2 (3.2)	1 (2)	5 (3.4)	4 (3.3)	2 (2.1)	NS	NS	NS	NS
CR1*AGAGTG	3 (1.5)	23 (5.2)	5 (2.8)	10 (6.5)	3 (7.9)	4 (6.4)	6 (12.5)	7 (4.7)	13 (12.7)	0	p = 0.035; 3.99 [1.10–14.15]	NS	p = 0.03; 5.51 [1.17–25.8]	p = 0.005; 7.77 [1.84–32.7]
CR1*AGGGTG	1 (0.5)	17 (3.9)	6 (3.4)	8 (5.2)	0	1 (1.6)	3 (6.2)	6 (4)	6 (5.9)	3 (3.2)	NS	p = 0.028; 12.15 [1.30–113]	NS	p = 0.037; 11.14 [1.15–107]
CR1*AGAAAA	2 (1)	8 (1.8)	3 (1.7)	3 (1.9)	2 (5.3)	0	0	3 (2)	0	3 (3.2)	NS	NS	NA	NA
CR1*AGAGTA	3 (1.5)	2 (0.5)	1 (0.6)	1 (0.6)	0	0	0	1 (0.7)	0	1 (1)	NS	NS	NS	NS
CR1*AAAATA	0	2 (0.5)	0	2 (1.3)	0	0	0	1 (0.7)	1 (1)	0	NA	NA	NA	NA
CR1*AAGAAG	0	1 (0.2)	0	0	0	1 (1.6)	0	1 (0.7)	0	1 (1)	NA	NA	NA	NA
CR1*GGGAAA	0	1 (0.2)	0	0	0	1 (1.6)	0	1 (0.7)	0	1 (1)	NA	NA	NA	NA
CR1*AAGATG	0	1 (0.2)	0	0	0	1 (1.6)	0	1 (0.7)	1 (1)	0	NA	NA	NA	NA
CR1*AGGATG	0	1 (0.2)	1 (0.6)	0	0	0	0	0	0	0	NA	NA	NA	NA
CR1*GGAGTG	0	1 (0.2)	1 (0.6)	0	0	0	0	0	0	0	NA	NA	NA	NA
CR1*AGGATA	1 (0.5)	0	0	0	0	0	0	0	0	0	NA	NA	NA	NA

NA: Not applicable.

NS: Not significant.

## Discussion

In order to maintain its life cycle after transmission by triatomine vectors to a human host, *T. cruzi* needs to evade host immune attack and develops further intracellularly. The successful entrance of *T. cruzi* into host cells depends on the down-regulation of complement activation by parasite regulatory molecules and by its binding to complement receptors such as CR1^10,11,24^. Thus, complement system and CR1 have an important role both in the establishment of *T. cruzi* infection and sustenance of the chronic phase. In this study, the *CR1* genetic variants in exon 29 were investigated in patients with chronic CD in order to assess their role in the modulation of CR1 levels as well as in the development and in the clinical progression of the disease.

Patients with chronic CD had significantly decreased levels of CR1 compared to healthy controls. Plasma levels observed in the control group were in accordance with those reported in other studies^38,40^. The reduced CR1 expression on erythrocytes combined with increased levels of immune complexes has been demonstrated in the pathogenesis of HIV, SARS-CoV, *M. tuberculosis* and *M. leprae* infections^33,36,37,39^. In leprosy, AIDS, and tuberculosis, the reduction of CR1 levels is disease regulated, demonstrating that this condition is acquired rather than inherited^36,39^. Moreover, it is known that, similarly to mechanisms used by other pathogens, *T. cruzi* uses C1q to promote C1-dependent phagocytosis as well as MBL and ficolin-2 to promote opsonization via CR1 as a strategy to evade the host immune system and infect host cells^15^.

Interestingly, patients with cardiomyopathy had lower CR1 plasma levels than asymptomatic patients, which might indicate either consumption due to increased complement activation or lower production associated with this clinical manifestation. In fact, chagasic cardiomyopathy is known to be associated with inflammatory process and tissue damage, as observed in various inflammatory and infectious conditions^41–44^. Moreover, one of the consequences of the persistent myocardial damage in CD is left ventricular dilation with systolic dysfunction^45^. For this reason, left ventricular systolic function was evaluated in CD patients using the Left Ventricular Ejection Fraction (LVEF). Despite the important role of the complement system in cardiovascular diseases^46,47^ such as atherosclerosis^48,49^, myocardial infarction^50,51^, and acute ischaemic stroke^52^, no correlation between CR1 levels and LVEF was found. This finding corroborates with data from a study on patients with acute myocardial infarction^53^.

It is known that CR1 levels may be influenced by infections and that their expression is associated with genetic as well as acquired factors^39^. It was observed in this study that lower levels of CR1 were associated with rs6691117*GG* genotype in the controls, but not in patients. Two other studies found this genotype associated with lower erythrocyte sedimentation rate^54^ and with preterm birth^55^. These findings indicate that rs6691117*GG* genotype may modulate CR1 expression. Since there was no association between the rs6691117*GG* and CR1 levels in the patients, the reduction of CR1 levels in chronic CD is probably due to the disease process. An anti-inflammatory role for CR1 was already observed in experimental studies where CR1 was able to prevent tissue injury induced by complement activation^56^. Considering that chronic CD is associated with inflammation, it is possible that the low levels of CR1 in CD patients may be related to its anti-inflammatory effect and consumption due to complement activation. However, the exact mechanism, which controls the expression of CR1 in CD patients is still unclear.

The positive association of *AG* and *GG* genotypes (in variants rs17047661, rs6691117) and the *G* allele (in variant rs17047660*G*) observed with chronic CD may be related to the functional properties of the CR1 molecule. These variants lead to the substitutions of amino acids in the CR1 molecule which may affect the folding and the affinity of CR1 to C3b, C4b and C1q/MBL/ficolin-2^17,20–22,57,58^. The allele rs6691117*G* was also related to a low ratio of CR1 expression in erythrocyte membranes^54^. In addition, the alleles rs17047660*G* and rs17047661*G* were previously associated with severe malaria^59^, sickle cell anemia^60^, and showed to have protective effects against *M. leprae*^30^ and *M. tuberculosis* infection^61^, while allele rs6691117*G* increased risk of Alzheimer disease^62^, gastric cancer^63^, non-small cell lung cancer^64^ and preterm birth^55^.

Moreover, the allele rs17047661*G* and CR1*AGGGTG and *AGAGTG* haplotypes were related to early stages of CD cardiac form indicating that these variants might predispose to clinical progression of chronic patients with CD. Since the pathogenesis of CCC involves parasite persistence in different tissues as well as continuous low-grade parasitemia, inflammatory process and immune mediated-myocardial injury, it is possible that protein products of these *CR1* variants may augment *T. cruzi* binding with consequent cellular internalization besides having an immunomodulatory effect.

A limitation of the present study is the lack of baseline CR1 plasma levels in patients with acute CD. Acute CD patients are difficult to diagnose clinically and hence the measurement of CR1 levels was not possible during their early stages of infection that might serve as a baseline measurement. The Ambulatory of Chagas Disease of Hospital das Clínicas (Federal University of Paraná) enrolls only chronic CD patients, thus making the access to acute CD patients impossible.

In conclusion, this study reports that *CR1* variants are associated with the risk of *T. cruzi* infection and to progression to chagasic cardiomyopathy. Besides that, the low of CR1 levels observed in CD patients is possibly due to the disease process. This is the first study that provides insights on the role of CR1 in development and clinical presentation of chronic CD. Nevertheless, further studies are necessary to confirm these findings.

## Methods

### Study Population

A total of 232 chronic CD patients attending at Ambulatory of Chagas Disease of Hospital das Clínicas, Federal University of Paraná, were investigated [mean age 57 years; 130 (56%) females, 102 (44%) males, 176 (75.9%) Euro-, 44 (19%) Afro-Brazilian, 1 (0.4%) Asian, 11 (4.7%) Amerindian]. CD diagnosis was performed by two serological tests (ELISA and immunofluorescent antibody assay). Clinical findings were in accordance with those outlined by the Pan-American Health Organization (PAHO) and World Health Organization (WHO)^2,65^. Clinical details of the patients were obtained through medical records and interviews. Patients younger than 18 years old with recent infection or suspected non-chagasic cardiomyopathy were excluded. Demographic and clinical characteristics of the distinct CD forms are shown in Table 4. Patients with cardiomyopathy were graded according to the cardiac insufficiency classification of the American Heart Association, adapted for CD^66^: **A**, altered electrocardiogram (ECG) and normal echocardiogram (ECHO), absence of cardiac insufficiency (CI); **B1**, altered ECG, LVEF > 45%, absence of CI; **B2**, altered ECHO, LVEF < 45%, absence of CI; **C**, altered ECG and ECHO, compensable CI; **D**, altered ECG and ECHO, refractory CI. A group of 104 healthy Brazilians [mean age 51 years; 50 (48.1%) females, 54 (51.9%) males, 91 (87.5%) Euro-, 10 (9.6%) Afro-Brazilian, 2 (1.9%) Asian, 1 (1%) Amerindian] was used as control. All individuals from the control group were selected consecutively from a blood bank in the same geographic region as patients. Following Brazilian health regulations, the blood donors were screened for CD, syphilis, hepatitis B, hepatitis C, HIV and human T-cell lymphotropic viruses 1 and 2 using high sensitivity assays. Additionally, information about autoimmune diseases and cancer background was obtained during the pre-selection interview^67,68^. The study protocol was approved by the Ethics Committee of the Hospital de Clínicas, Federal University of Paraná (CEP/HC-UFPR n. 360.918/2013-08), and performed in accordance with relevant guidelines/regulations. Written informed consent was obtained from all patients and controls.

**Table 4:** Baseline clinical parameters of the investigated study cohort.

	Indeterminate (n = 92)	Cardiac (n = 87)	Digestive (n = 21)	Cardiodigestive (n = 32)	Controls (n = 104)
Age [Range]	57 [34–76]	51 [34–90]	57 [36–81]	57 [37–73]	51 [37–72]
Sex (Male/Female)	34/58	46/41	15/16	18/14	54/50
Ethnicity (Euro-Brazilian/Others)	80/12	58/29	15/6	23/9	91/13
Cardiac impairment (A,B,C,D)*	NA	(27,22,36,02)	NA	(11,07,12,02)	NA
Erythrocytes (RBCs) (Million cells/µL), [Range]	4.7 [4.2–6.5]	5.0 [3.6–6.0]	4.8 [4.2–6.5]	5.0 [4.3–5.6]	NA
Hemoglobin (mg/dL), [Range]	14.4 [10.9–45.7]	14.8 [9.0–17.7]	14.6 [13.2–17.3]	14.9 [12.9–17.4]	NA
uCRP levels (mg/dL), [Range]	0.33 [0.08–3.77]	0.34 [0.08–4.25]	0.19 [0.09–0.38]	0.34 [0.08–0.76]	NA
CR1 levels (ng/mL), [Range]	11.73 [6.16–51.61]	10.72 [6.16–37.93]	9.46 [6.74–53.17]	9.64 [7.34–40.97]	17.25 [6.69–67.35]
LVEF (%), [Range]	70 [35–84]	65 [45–82]	NA	66 [47–77]	NA

NA: Not applicable.

*Cardiac patients were graded according to the cardiac insufficiency classification of the American Heart Association (AHA) adapted for CD.

RBCs: Red blood cells.

uCRP: Ultrasensitive C-reactive protein.

CR1: Complement receptor 1.

LVEF: Left ventricular ejection fraction.

### CR1 genotyping

In order to assess the distribution of the six functional *CR1* exon 29 variants rs17259045 (g.207609362 A > G, p.N1540S), rs41274768 (g.207609424 G > A, p.V1561M), rs17047660 (g.207609511 A > G, p.K1590E), rs17047661 (g.207609544 A > G, p.R1601G), rs4844609 (g.207609571 T > A, p.T1610S) and rs6691117 (g.207609586 A > G, p.I1615V), the entire *CR1* exon 29 including its intron-exon boundaries was directly sequenced only in 220 patients with chronic CD and 102 healthy control individuals. DNA from 12 patients and from two controls was degraded; therefore these individuals were excluded from further genetic analyses. Genomic DNA was extracted from buffy-coats using the QIAamp Blood mini kit (Qiagen GmbH, Hilden, Germany) following the manufacturer’s instructions. The *CR1* reference sequence was retrieved from the Ensembl database (www.ensembl.org); primers targeting exon 29 of *CR1* gene were designed manually, tested using Primer-BLAST (http://www.ncbi.nlm.nih.gov/tools/primer-blast) and synthesized commercially (Eurofins Genomics, Ebersberg, Germany). A fragment of 884 bp was amplified by polymerase chain reaction (PCR) using the *CR1* locus specific primer pair CR1F (5′-TCT TCA TAA ATA ATG CCA GAA GTG G-3′) and CR1R (5′-TGC CAA TTT CAT AGT CCT TAT ACA C-3′). PCR amplifications were carried out in a 25 µl volume of reaction mixture containing 10 × PCR buffer, 3.0 mM MgCl2, 0.2 mM dNTPs, 0.2 µM of each primer, 1 unit of Taq polymerase (Qiagen) and 20 ng of genomic DNA on a TProfessional Basic Thermocycler (Biometra GmbH, Göttingen, Germany). Cycling parameters were initial denaturation at 94 °C for 5 minutes, followed by 40 cycles of denaturation at 94 °C for 30 seconds, annealing at 55.5 °C for 30 seconds and elongation at 72 °C for 1 minute, and a final elongation step at 72 °C for 10 minutes. PCR fragments were stained with SYBR Safe DNA Gel Stain (Invitrogen, Carlsbad, USA) and visualised in a 1.5% agarose gel. PCR products were purified using Exo-SAP-IT (USB-Affymetrix, Santa Clara, USA) and the purified products were directly used as templates for sequencing using the BigDye terminator cycle sequencing kit (v.3.1; Applied Biosystems, Texas, USA) on an ABI 3130XL DNA Analyzer. DNA polymorphisms were identified by assembling the sequences with the reference sequence of the *CR1* (NM_000573) using the Geneious v9.1.4 software (Biomatters Ltd, Auckland, New Zealand) and reconfirmed visually from their respective electropherograms.

### Quantification of soluble CR1 plasma levels

Measurements of erythrocyte CR1 plasma levels were performed in 221 patients and 102 controls using a commercial high-sensitivity ELISA kit (Human Complement Receptor 1/SEB123Hu, Cloud-Clone Corporation, Texas, USA) in accordance with the manufacturer’s instructions. The limit of detection was 0.312 ng/mL.

### Statistical Analysis

CR1 plasma levels were compared between groups using nonparametric Kruskal-Wallis and Mann-Whitney tests. The distribution of each variable was assessed by the Shapiro-Wilk test. Multiple logistic regression was executed with adjustment for age, sex, and ethnic group. Multiple comparisons were corrected using a Benjamini-Hochberg procedure applying a false discovery rate of 0.10 and raw *p*-values that remained significant after this correction were considered in the study. Odds ratios (OR) and their 95% confidence intervals (CI) were calculated using the STATA software (v. 12.0, StataCorp, College Station, Texas, USA). Correlation analyses were performed by non-parametric Spearman’s rank coefficient tests. Allele frequencies were obtained by direct counting. Genotype and haplotype frequencies were analyzed by gene counting and expectation-maximum (EM) algorithms and the significance of deviation from Hardy-Weinberg equilibrium was tested using the random-permutation procedure as implemented in the Arlequin v. 3.5.2.2 software (http://lgb.unige.ch/arlequin). Linkage disequilibrium (LD) analysis was performed using Haploview v. 3.2 (http://broadinstitute.org/haploview). Possible associations of *CR1* alleles, genotypes, and haplotypes with different clinical forms were evaluated with two-tailed Fisher exact tests. *P-*values < 0.05 were considered significant.
